# Dietary long chain n-3 polyunsaturated fatty acid induced regulatory T-cells contribute to the prevention of oral sensitization to cow’s milk protein in mice

**DOI:** 10.1186/2045-7022-3-S3-O18

**Published:** 2013-07-25

**Authors:** L Van Den Elsen, B van Esch, G Hofman, B van de Heijning, J Garssen, L Willemsen

**Affiliations:** 1 Utrecht Institute for Pharmaceutical Sciences, Utrecht University, Utrecht, the Netherlands; 2 Danone Research, Wageningen, the Netherlands; 3 Utrecht University, Utrecht, the Netherlands

## Background

Cow’s milk allergy is one of the most common food allergies in children. Dietary lipid composition may affect the susceptibility to develop allergic diseases. Aim of this study was to assess whether and how dietary supplementation with long chain n-3 polyunsaturated fatty acids (n-3 LCPUFA) prevents the establishment of food allergy.

## Methods

Donor mice were fed a control or fish oil diet before and during oral sensitization with whey. Acute allergic skin response, serum immunoglobulins as well as dendritic cell (DC) and T-cell subsets in mesenteric lymph nodes (MLN), spleen and/or small intestine were assessed. Besides serum transfer experiments, adoptive transfer experiments with splenocytes of whey-sensitized donor mice fed a control or fish oil diet were performed. For this purpose naïve recipient mice received splenocytes either or not *ex vivo* depleted of CD25+ cells, or MACS isolated CD4+CD25+ regulatory T-cells (Treg). Recipient mice were sham- or whey-sensitized and fed control diet.

## Results

The acute skin response as well as whey-IgE and -IgG1 levels were reduced in sensitized donor mice fed the fish oil diet as compared to the control diet. Serum transfer confirmed the Th2 type humoral response to be suppressed since sera of fish oil fed sensitized mice had a diminished capacity to induce an allergic response in naïve recipient mice compared to control sera. Furthermore, the acute skin response was diminished upon passive sensitization with hyperimmune serum in fish oil fed naïve recipient mice indicating suppression of the effector response by n-3 LCPUFA. Fish oil fed whey-sensitized donor mice showed an increased % Treg inducing CD11b+CD103+CD8α- DC in MLN in association with enhanced FoxP3+ Treg in spleen and intestine compared to sham mice. In whey-sensitized recipient mice transferred with splenocytes from whey-sensitized fish oil fed donor mice, the acute allergic skin response was similar to sham-sensitized recipients. *Ex vivo* depletion of Treg prevented this transfer of tolerance. Transfer of CD4+CD25+ Treg (85% was FoxP3+) from fish oil fed whey sensitized donors prevented the acute allergic skin response most pronounced.

## Conclusion

Dietary n-3 LCPUFA largely prevent allergic sensitization in a mouse model for cow’s milk allergy by suppressing the humoral response, enhancing local and systemic Treg and reducing acute allergic symptoms. FoxP3+ Treg play an important role in whey allergy prevention by n-3 LCPUFA.

## Disclosure of interest

L Van Den Elsen: None declared, B van Esch: Employee of Danone Research, G Hofman: None declared, B van de Heijning: Employee of Danone Research, J Garssen: Employee of Danone Research, L Willemsen: None declared.

